# Gap between desired and self-determined roles of general practitioners: a multicentre questionnaire study in Japan

**DOI:** 10.1186/s12875-021-01512-x

**Published:** 2021-07-31

**Authors:** Takashi Chinen, Yusuke Sasabuchi, Kazuhiko Kotani, Hironori Yamaguchi

**Affiliations:** 1 Department of Clinical Oncology, Jichi Medical University, 3311-1 Yakushiji, Shimotsuke-Shi, Tochigi 329-0498 Japan; 2 Data Science Center, Jichi Medical University, Shimotsuke-Shi, Tochigi, 329-0498 Japan; 3 Department of Community Medicine, Jichi Medical University, Shimotsuke-Shi, Tochigi, 329-0498 Japan

**Keywords:** General practitioners, Neoplasms, Palliative care, Cancer survivors, Primary health care, Surveys and questionnaires

## Abstract

**Background:**

Primary care physicians have diverse responsibilities. To collaborate with cancer specialists efficiently, they should prioritise roles desired by other collaborators rather than roles based on their own beliefs. No previous studies have reported the priority of roles such clinic-based general practitioners are expected to fulfil across the cancer care continuum. This study clarified the desired roles of clinic-based general practitioners to maximise person-centred cancer care.

**Methods:**

A web-based multicentre questionnaire in Japan was distributed to physicians in 2019. Physician roles within the cancer care continuum were divided into 12 categories, including prevention, diagnosis, surgery, follow-up with cancer survivors, chemotherapy, and palliative care. Responses were evaluated by the proportion of three high-priority items to determine the expected roles of clinic-based general practitioners according to responding physicians in similarly designated roles.

**Results:**

Seventy-eight departments (25% of those recruited) from 49 institutions returned questionnaires. Results revealed that some physicians had lower expectations for clinic-based general practitioners to diagnose cancer, and instead expected them to provide palliative care. However, some physicians expected clinic-based general practitioners to be involved in some treatment and survivorship care, though the clinic-based general practitioners did not report the same priority.

**Conclusion:**

Clinic-based general practitioners prioritised involvement in prevention, diagnoses, and palliative care across the cancer continuum, although lower expectations were placed on them than they thought. Some additional expectations of their involvement in cancer treatment and survivorship care were unanticipated by them. These gaps represent issues that should be addressed.

## Background

Cancer is a substantial health problem and the first or second leading cause of middle-aged death worldwide [1]. The care of cancer patients includes multiple complex health problems in each phase of the cancer care trajectory [2, 3]. From primary prevention, diagnosis and treatment to palliative care, physicians of various specialties perform specific roles for cancer control [2, 3]. Among them, primary care physicians can demonstrate their strengths in particular for prevention and diagnosis, survivorship care, shared follow-up care, and palliative care [3]. Hence, without integration of the cancer care and communication between primary care physicians and cancer specialists, the care can be fragmented and of poor quality [2, 4–7].

Several previous studies have reported on the crucial services provided by general practitioners (GPs) in the context of cancer care, especially regarding the need for collaboration with oncologists [8–11]. In addition, numerous interviews and questionnaires have been conducted with oncologists [12], GPs [13–15], and patients [16, 17], and some studies have shown that home-based palliative care is dependent upon GP competency [18]. Additionally, research has indicated that cancer survivors benefit from shared care provided by GPs who assist oncologists [19]. On the other hand, GPs have assumed growing responsibilities for various healthcare services in addition to cancer care that can place heavy burdens on them [20, 21], especially in rural areas due to few healthcare resources [11]. For GPs to efficiently collaborate with cancer specialists to maximise total person-centred cancer care, they should prioritise and select the roles desired by other collaborators rather than rely on their own beliefs about their roles across the cancer care continuum.

**Fig. 1:**
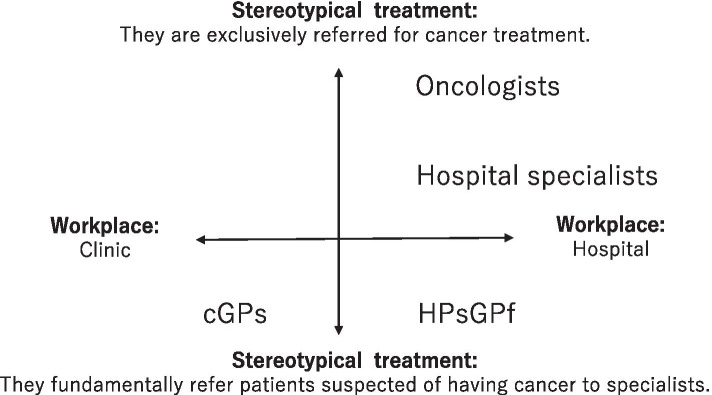
Physicians involved in cancer treatment divided by title. HPsGPf = Hospital-based physicians with general practitioner function; cGPs = clinic-based general practitioners

In Japan, primary healthcare services are provided in both community clinics with limited amounts of inspection equipment and the outpatient departments of smaller-scale private hospitals in general [22]. For that reason, this study categorised GPs into two types: 1) clinic-based GPs (i.e. primary care providers; PCPs), and 2) hospital-based physicians who also have GP functions. In addition to these two types of GPs and oncologists, other hospital specialists with departmental expertise are generally responsible for managing nearly all inpatient and outpatient care based on the specifically affected organ. While these doctors are not as specialised in treating cancer as oncologists, they may also provide palliative care in some cases. In sum, four types of relevant physicians were chosen for the context of this study: oncologists, hospital specialists, hospital-based physicians with GP functions, and clinic-based GPs, each of whom are active across the cancer care continuum (Fig. 1). Indeed, many physicians in Japan work together to develop medical services directed at treating cancer throughout the country. In this regard, the Japanese government approved the Cancer Control Act in 2006 and launched the Basic Plan to Promote Cancer Control Programs in 2007 [23].

No previous studies have reported the priority of tasks clinic-based GPs are expected to fulfil when collaborating with cancer specialists across the cancer care continuum. This study investigated the roles expected to be filled by clinic-based GPs across the cancer care continuum based on the opinions of four types of priority physicians. In this regard, the primary aim of this study was to clarify the roles clinic-based GPs should prioritise in order to better explore how physicians should unite and cooperate to effectively share responsibilities in cancer care as one team.

## Methods

### Aim

The aim of this manuscript is to clarify clinic-based general practitioners’ roles desired by collaborators across the cancer care continuum.

### Study design

This was a web-based multicentre questionnaire study.

### Setting, participants, and data sources

Data were collected from February to June 2019. Specifically, this study’s researchers sent e-mails explaining the purpose of the study and including the URL for accessing the questionnaire to each medical department of facilities belonging to the Japan Association for Development of Community Medicine (JADECOM), which is a public interest incorporated association that aims to secure community medicine in Japan. Participating facilities included 24 hospitals, 33 clinics, 7 geriatric health service facilities and 10 complex facilities across Japan [24]. In each facility, one physician working in the medical department and one additional full-time physician were eligible to answer one questionnaire each to reduce selection bias. Among all participating facilities, 311 departments were included. No respondents were given incentives to complete the questionnaire, and the managers of each facility sent reminder e-mails to non-respondents at 1- and 3-month intervals after initially sending out the invitation. Incomplete questionnaires were not accepted.

### Questionnaire

The questionnaire contained the following four sections:

1. Demographic and professional characteristics of the responding physicians: facility, department, age, whether they were involved in cancer care, and hospital physician status (respondents of hospital physician chose one physician type of the three available here).
2. From the following list of 12 provided roles, respondents chose the three they considered to be the highest priority in the cancer care continuum for themselves.
  1. Primary cancer prevention (total health promotion, including recommending smoking cessation, drinking moderation, and lifestyle habits)
  2. Secondary prevention (medical examinations and early diagnosis)
  3. Administering advice regarding total cancer care, depending upon patient requests
  4. Surgery (including endoscopic resection)
  5. Radiation therapy
  6. Outpatient follow-up appointments to identify potential cancer recurrence after local treatment
  7. Chemotherapy as oncologists
  8. Chemotherapy managed by oncologists from other hospitals
  9. Management of the adverse effects of chemotherapy
  10. Somatic pain control
  11. Psychological support
  12. Home medical care
3. Questions regarding specific actions in cancer care: surgery and endoscopic resection, radiotherapy, chemotherapy, and palliative care. More specifically, participants responded to items concerning the percentage of patients diagnosed with cancer they expected would receive treatment in their facilities, what kinds of cancer they typically treated, whether they continued medical treatments after referring cancer patients to cancer centres, and whether they took measures for immune-related adverse events (irAEs).
4. From the list of 12 provided roles, respondents chose the three that they considered to be the highest priority for clinic-based GPs in the cancer care continuum.

Regarding the roles of physicians in the cancer care continuum, respondents were asked to prioritise the 12 items twice. The first prioritisation was to assess their own roles, while the second was to provide their opinions on the expected roles of clinic-based GPs. Between the two times, the questions mentioned above were asked about specific cancer care to provide an overview of the cancer care continuum.

### Variables

The top three priority items believed about themselves were tabulated, then the proportions of the selected items based on the four types of physicians were calculated. This allowed researchers to compare the expected roles of the four types of physicians.

The same procedure was then used to calculate the top three priority items expected about clinic-based GPs. Finally, the ratios of expectations of clinic-based GPs by the other three types of cancer care physicians were compared to the self-believed roles reported by the clinic-based GPs themselves.

### Statistical methods

The Kruskal–Wallis test was used to test for differences between the four groups (significance level set to *p* < 0.05). All analyses were conducted using R V.4.0.4 (The R Foundation, Vienna, Austria). There were no missing data because of the above collection process.

## Results

A total of 78 departments (25% of all those recruited) from 49 institutions returned completed questionnaires. Table 1 shows a breakdown of their institutional characteristics, and Table 2 shows their specific characteristics. As shown in Table 2, there were 11 oncologists (i.e. six gastrointestinal and general surgeons, and one oncologist each in internal medicine, respiratory medicine, otorhinolaryngology, dermatology, and psychiatry). There were 22 hospital-based physicians with GP functions and 15 clinic-based GPs. Of the 13 physicians who were unrelated to cancer care, there were six clinic-based and/or geriatric health care facilities-based GPs and seven hospital physicians (two ophthalmologists, two cardiologists, one cardiovascular surgeon, one orthopaedic surgeon and one paediatrician).

**Table 1:** Participant recruitment, 2019

Institutional characteristics	n (%)
Hospitals	18 (75)
Clinics	20 (61)
Geriatric health service facilities	3 (43)
Clinic and geriatric health care complexes	8 (80)
Total institutions	49 (66)
Total responding departments among total departments recruited from all institutions	78 (25)

**Table 2:** Participant demographic characteristics, 2019

Physician characteristics	n (%)
Hospital physicians	47 (60)
Clinics and geriatric health facilities physicians	31 (40)
Ages	
20–29	1 (1)
30–39	7 (9)
40–49	24 (31)
50–59	31 (40)
≥ 60	15 (19)
Physician type	
Oncologists	11 (14)
Hospital specialists	7 (9)
Hospital-based physicians with GP functions	22 (28)
Clinic-based GPs	15 (32)
Physicians unrelated to cancer care	13 (17)

*GP* General practitioner

**Fig. 2:**
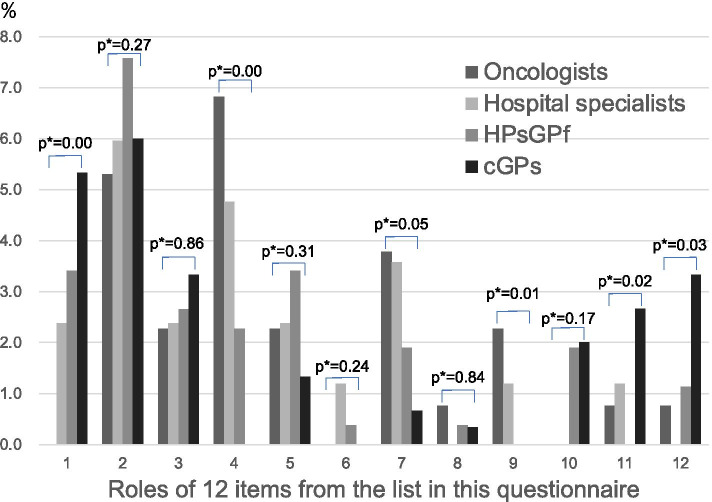
Proportions of each physician’s self-believed roles in cancer care, 2019. HPsGPf = Hospital-based physicians with general practitioner function; cGPs = clinic-based general practitioners. p* (*p*-values were calculated using the Kruskal–Wallis test). To calculate these proportions, first, we counted the top three selected priority items. Second, we divided the count of each item by three times the number of each physician. Third, we divided the ratios by four and multiplied by 100, for the total of four physicians, which totalled the ratios of each item to 100%. Finally, to compare these proportions of each physician by item, we arranged the proportions of the four physicians side by side for each item

Figure 2 shows the proportions of each physician role based on the 12 listed roles. Clinic-based GPs tended to be involved in prevention, examinations, and diagnoses of cancer, and to provide palliative care as in numbers 10, 11, and 12. No clinic-based GPs selected number 4 ‘surgery and endoscopic resection’, number 6 ‘outpatient follow-up appointments for cancer recurrence after local treatment’, or number 9 ‘managing the adverse effects of the chemotherapy’. The Kruskal–Wallis test results indicated that the proportions were not similar for numbers 1, 4, 9, 11 and 12. This means that clinic-based GPs fulfilled more responsibilities in ‘primary cancer prevention’, ‘psychological support’, and ‘home medical care’ across the cancer continuum and less responsibilities in ‘surgery and endoscopic resection’ and ‘managing the adverse effects of the chemotherapy’.

Further, some clinic-based GPs selected numbers 7 and 8 (about chemotherapy); four clinic-based GPs provided chemotherapy for prostate cancer, two did so for breast cancer, and one did so for brain tumours and pancreatic cancer. No institution with clinic-based GPs took measures for irAEs. The percentages of institutions with oncologists and other hospital physicians were 54.5% and 17.2%, respectively.

Hospital-based physicians with GP functions were more likely to select number 2 ‘medical examinations and early diagnosis’ than were clinic-based GPs but less likely to choose number 11 ‘psychological support’.

**Fig. 3:**
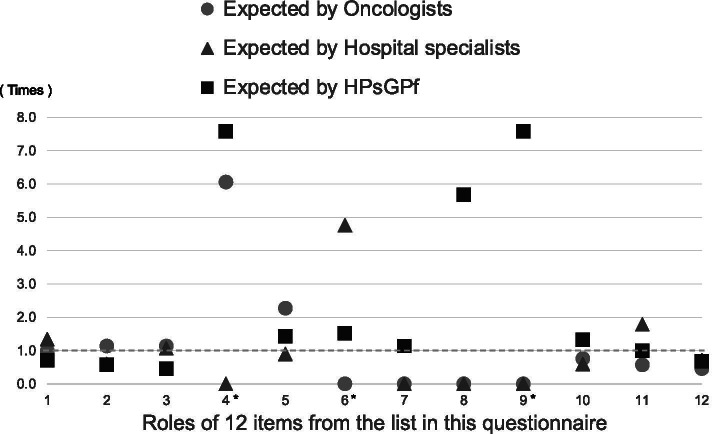
Ratios of clinic-based general practitioners’ roles expected by collaborators to clinic-based general practitioners’ self-believed roles. HPsGPf = Hospital-based physicians with general practitioner function. To calculate these ratios, we divided the proportion of expected roles of clinic-based general practitioners according to the other three types of cancer care physicians by the proportion of roles reported by the clinic-based general practitioners. For 4*, 6*, and 9*, the denominator, which is the proportion of self-believed roles formerly filled by clinic-based general practitioners in Fig. 2, was assumed to be one because calculating with zero becomes infinite

Figure 3 shows the ratios of roles expected of clinic-based GPs according to the other three types of cancer care physicians to the self-believed roles fulfilled by the clinic-based GPs. With regard to numbers 1, 2, 10, 11, and 12 (about prevention, diagnosis, and palliative care), more clinic-based GPs selected them as roles they believed that they prioritised; hospital specialists were more likely to expect clinic-based GPs to be involved in number 11 ‘psychological support’ but less likely to expect them to be involved in diagnoses and home medical care than the clinic-based GPs reported.

Regarding numbers 4, 6, and 9, as no clinic-based GPs selected these as their priority, a ratio was impossible (with a denominator of 0); therefore, we calculated this ratio with the denominator set to one. With that in mind, some oncologist and hospital-based physicians with GP functions expected clinic-based GPs to be involved in ‘surgery and endoscopic resection’ (number 4), some hospital specialists expected clinic-based GPs to be involved in ‘outpatient follow-up appointments for cancer recurrence after local treatment’ (number 6), and some hospital-based physicians with GP functions expected clinic-based GPs to be involved in ‘chemotherapy managed by oncologists from other hospitals’ (number 8) and ‘managing the adverse effects of the chemotherapy’ (number 9).

## Discussion

### Key findings

This study investigated the gap between expected and self-determined roles of clinic-based GPs across the cancer care continuum in Japan to clarify what clinic-based GPs should prioritise. Results showed that some physicians had lower expectations that clinic-based GPs would diagnose cancer and provide palliative care than the clinic-based GPs believed. On the other hand, some responding physicians expected clinic-based GPs to be involved in treatment and survivorship care, which clinic-based GPs did not prioritise.

### Strengths and limitations

This is the first study to explore GPs’ roles across the cancer care continuum according to the roles other physicians expected, aiming to unite those involved in cancer care as one team so they could cooperate and effectively share responsibilities. In this way, we can clarify the issues that should be prioritised in the healthcare system.

This study had some limitations. First, there was a selection bias in the recruiting process, as the proportions of hospital physicians and those working at clinics and/or geriatric health care facilities were 60% and 40%, respectively. However, a 2016 survey conducted by the Ministry of Health, Labour and Welfare revealed that these proportions were 65.7% and 34.4%, respectively [25]. Second, the sample size was small because of the low response rate. Third, the 12 items listed on the questionnaire, which divided roles across the cancer continuum, were not validated, although they were established by replicating previous studies [2, 3]. Fourth, the division of hospital physicians related to cancer care into three types was not validated. Fifth, some clinics in Japan are able to provide esophagogastroduodenoscopies and computed tomography scan examinations. Thus, it would be difficult to generalise the result of this study to any hospital without that capability. Further studies are required to discern which roles of GPs best serve patient outcomes across the cancer care continuum.

### Comparison to existing literature

In this study, each physician type extended the highest priority to examinations and diagnoses (some hospital physicians who highly prioritised cancer diagnoses did not expect clinic-based GPs to do so). Notably, a previous study found that some clinic-based GPs delayed cancer diagnoses through inappropriate screenings [26]. Clinic-based GPs should keep in mind that sometimes it is better to deprioritise cancer diagnoses and entrust them to hospital-based GPs. To achieve the timely diagnosis of cancer, several approaches have previously been suggested: patients’ education, continuing GPs’ education, sufficient communication with specialists, improving the health care system, and so on [27].

Some responding physicians expected clinic-based GPs to be involved in surgery (including endoscopic resection), although clinic-based GPs did not prioritise this action. Although this finding is difficult to explain, it may be that some responding physicians want to show they can perform endoscopic resection in some primary care settings.

This study supports previous research findings that clinic-based GPs were expected to conduct follow-up appointments with cancer survivors [2, 19]. However, the clinic-based GPs themselves did not prioritise this role. In Japan, a cancer survivorship guideline is going to be established to coordinate care between oncologists and GPs [28, 29]. The results of this study indicate that cancer survivor follow-up was a burden to hospital specialists and some of them wanted to share this responsibility with GPs if possible.

Some responding physicians expected clinic-based GPs to be involved in chemotherapy; in actuality, clinic-based GPs may also help patients with prostate and breast cancers through hormonal therapies [30]. Although clinic-based GPs did not intend to get involved in ‘the management of adverse effects of chemotherapy’ as a priority, with regard to irAEs, it is important to note that new cancer immunotherapies may produce new adverse events in the form of various types of symptoms and diseases [31]. All physicians should be aware that immunotherapies are becoming increasingly popular for treating cancer patients.

Clinic-based GPs were expected to be involved in palliative care as other physicians do not engage in this work (some physicians did not expect clinic-based GPs to do so). Previous studies have shown that palliative care needs are present during the chemotherapy phase in the cancer trajectory [32] and in the context of home-based care [19]. This study supports the current literature that states that clinic-based GPs should be expected to perform duties at the entrance and exit of cancer care, meaning that few duties should be expected from them between those two points with shared follow-up care. These GPs are specifically expected to complete tasks in palliative care and during the post-chemotherapy phase even if they do not actually administer anti-cancer agents. The results of this study further reveal that some oncologists believe that performing duties at the entrance and exit of cancer care is too narrow, and that organised homebased specialist palliative-care teams can improve the overall cancer care process [33, 34]. Clinic-based GPs should also be aware of any patient concerns regarding chemotherapy treatments, such as the overall regimen and common side effects. If cancer patients have no pre-existing health conditions, then clinic-based GPs should be minimally involved during the chemotherapy stage regarding palliative care consultations. In Japan, fewer than 50% of clinic-based GPs are involved with palliative care [35, 36]. However, regional-based palliative care interventions have been shown to effectively improve cancer care treatments [37, 38]. In an educational approach, hospital physicians have successfully invited inexperienced doctors to accompany them at home visits designed to provide short-term palliative care, thus enabling them to learn directly about patient-centred and home-based care [39].

## Conclusions

Clinic-based GPs first prioritised preventions, diagnoses, and palliative care across the cancer care continuum, but others did not expect them to prioritise these same roles. At the same time, they did not expect other physicians to expect clinic-based general practitioners to be involved cancer treatment and survivorship care. These discrepancies are issues to be managed.

## Data Availability

No additional data are available.

## Abbreviation

GP: General practitioner
